# Another Pioneer Gone

**Published:** 1888-10

**Authors:** 


Another pioneer has gone: Dr. O. P. Baer, of Richmond, Indiana; born August 25th, 1816; died August 10th, 1888. He was the first homœopathic physician to make his home in Indiana. He practiced as an old-school physician ten years before becoming a homœopath.—*Recorder.*

